# Place of Death Among Decedents With Serious Mental Illness: The Role of Dementia and Family Availability

**DOI:** 10.1093/geroni/igaf122.029

**Published:** 2025-12-31

**Authors:** Malek Alnajar, Caroline Stephens, Eli Iacob, Djin Tay, Katherine Ornstein, Laura Block

**Affiliations:** University of Utah, Salt Lake City, Utah, United States; University of Utah, Salt Lake City, Utah, United States; University of Utah, Salt Lake City, Utah, United States; University of Utah, Salt Lake City, Utah, United States; Johns Hopkins University, Baltimore, Maryland, United States; University of Wisconsin-Madison, Madison, Wisconsin, United States

## Abstract

Older adults with serious mental illness (SMI), including major depression, schizophrenia, bipolar, and generalized anxiety, face complex health and social challenges (e.g., lack of family) that significantly impact their end-of-life experiences, including place of death. Yet, no study has comprehensively examined their end-of-life (EOL) experience. This retrospective cohort study compared place of death across major SMI conditions among individuals aged 55+ and described presence of comorbid dementia, family availability, and social determinants. Using data from the Utah Caregiving Population Science (Utah C-PopS) study, we analyzed 31,193 decedents with SMI. The majority were female (60.8%), non-Hispanic White (93.8%) with a mean age at death of 77.2 years (SD = 10.68). Major depression was most common (52.4%), followed by multiple SMI (21.7%), anxiety (20.7%), schizophrenia (2.7%), and bipolar (2.1%); one-third had comorbid dementia. Decedents with schizophrenia or co-occurring dementia were most likely to die in a long-term care facility (49.5% and 50.8%). Those with schizophrenia had the lowest family availability (28.3% with none) and a younger average age at death (mean=73.29 years), as did those with bipolar disorder (mean=72.67 years). Both groups were also more likely to be dual eligible and die in an urban setting. Individuals with multiple SMIs had the highest risk of multiple hospitalizations at EOL (47.2%). Findings highlight EOL challenges for older adults with SMI, especially those with schizophrenia and bipolar disorder, likely due to dementia burden and limited family support. Such insights can guide interventions to improve community support, hospice access, and family-centered care for older adults with SMI.

